# Transcripts Encoding the Androgen Receptor and IGF-Related Molecules Are Differently Expressed in Human Granulosa Cells From Primordial and Primary Follicles

**DOI:** 10.3389/fcell.2018.00085

**Published:** 2018-08-10

**Authors:** Line L. Steffensen, Emil H. Ernst, Mahboobeh Amoushahi, Erik Ernst, Karin Lykke-Hartmann

**Affiliations:** ^1^Department of Biomedicine, Aarhus University, Aarhus, Denmark; ^2^The Fertility Clinic, Horsens Hospital, Horsens, Denmark; ^3^The Fertility Clinic, Aarhus University Hospital, Aarhus, Denmark; ^4^Department of Clinical Medicine, Aarhus University, Aarhus, Denmark; ^5^Department of Clinical Genetics, Aarhus University Hospital, Aarhus, Denmark

**Keywords:** human granulosa cells, transcriptome, follicle development, AR, IGF

## Abstract

Bidirectional cross talk between granulosa cells and oocytes is known to be important in all stages of mammalian follicular development. Insulin-like growth factor (IGF) signaling is a prominent candidate to be involved in the activation of primordial follicles, and may be be connected to androgen-signaling. In this study, we interrogated transcriptome dynamics in granulosa cells isolated from human primordial and primary follicles to reveal information of growth factors and androgens involved in the physiology of ovarian follicular activation. Toward this, a transcriptome comparison study on primordial follicles (*n* = 539 follicles) and primary follicles (*n* = 261 follicles) donated by three women having ovarian tissue cryopreserved before chemotherapy was performed. The granulosa cell contribution in whole follicle isolates was extracted *in silico*. Modeling of complex biological systems was performed using IPA® software. We found the granulosa cell compartment of the human primordial and primary follicles to be extensively enriched in genes encoding IGF-related factors, and the Androgen Receptor (AR) enriched in granulosa cells of primordial follicles. Our study hints the possibility that primordial follicles may indeed be androgen responsive, and that the action of androgens represents a connection to the expression of key players in the IGF-signaling pathway including IGF1R, IGF2, and IGFBP3, and that this interaction could be important for early follicular activation. In line with this, several androgen-responsive genes were noted to be expressed in both oocytes and granulosa cells from human primordial and primary follicle. We present a detailed description of *AR* and *IGF* gene activities in the human granulosa cell compartment of primordial and primary follicles, suggesting that these cells may be or prepare to be responsive toward androgens and IGFs.

## Introduction

Female fertility is dependent on continuous (monthly) activation of primordial follicles from the resting dormant follicle pool. A primordial follicle is made up by an oocyte surrounded partly by flattened granulosa cells. As the primordial follicle is activated, it transforms into a primary follicle, where the oocyte is now surrounded by a complete layer of cubical granulosa cells. The primordial to primary follicle transition is a delicate and tightly regulated balance between activating and inhibiting factors with contribution from numerous different molecular pathways, but the mechanisms are not yet completely understood (Wandji et al., 1996). Bidirectional communication between the somatic granulosa cells and the oocyte is a fundamental part of both dormancy and activation, as well as the establishment of an optimal intrafollicular microenvironment (Eppig, 2001).

The essential role of androgens in normal ovarian function has been recognized for decades. Androgens play a key role by being the precursor of estradiol, however increasing evidence emphasize that the direct actions of androgens likewise are central for normal follicular development (Lebbe and Woodruff, 2013). It has been suggested that androgen sensitivity in early pre-antral follicles influence the primordial follicle recruitment (Vendola et al., 1999a; Stubbs et al., 2005; Yang et al., 2010). In the intraovarian communication, androgens may play a necessary role (Lebbe and Woodruff, 2013; Gervasio et al., 2014). Androgens bind the androgen receptor (AR) and exert the classical androgen response by genomic induction of transcription of several genes including *AR* itself, creating an autocrine loop between ligand and receptor (Weil et al., 1998; Gelmann, 2002). Besides the direct genomic effects, androgen signaling is also known to induce rapid non-genomic pathways via cytosolic AR and the mitogen-activated protein kinase extracellular signal-related kinase (MAPK/ERK) pathway (Kousteni et al., 2001). A balanced androgen level is however crucial, and exposure to excess androgens is associated with ovarian dysfunction. A large group of women suffering from ovarian dysfunction is women suffering from polycystic ovary syndrome (PCOS), a common endocrine disorder, in which hyperandrogenism is a key feature (Franks, 1995). Morphologically, polycystic ovaries have an increased percentage of growing follicles and “stockpiling” of the primary follicles compared to controls (Webber et al., 2003; Maciel et al., 2004). Moreover, clinical evidence from women exposed to androgen excess due to congenital adrenal hyperplasia (Hague et al., 1990) or exogenous testosterone treatment in female-to-male transsexuals (Spinder et al., 1989; Becerra-Fernández et al., 2014) underlines this picture by increased prevalence of morphologically polycystic ovaries compared to controls. Polycystic ovaries are also a common trait in prenatally androgenized sheep, an animal model for PCOS (Padmanabhan and Veiga-Lopez, 2013). Lambs born to dihydrotestosterone (DHT) or testosterone treated ewes showed the same pattern of dysfunctional early follicular development as the women suffering from PCOS. These examples emphasize the involvement of androgens in the early follicular development. In this follicular-phase gonadotropins are not obligatory, while local growth factors may play an important role. Insulin-like growth factor (IGF) signaling is a prominent candidate and may be connected to androgen-signaling. In the human ovary both IGF1 and IGF2 act as ligands for IGF receptor 1 (IGF1R) (Willis et al., 1998), and IGF2 expression is more prominent compared to other species (Mazerbourg et al., 2003). Rhesus monkeys treated with testosterone showed an increase in the fraction of activated primary follicles and a 5-fold increase in IGF1R mRNA in the oocytes of primordial follicles, as well as an elevation in the intra-oocyte IGF1 signaling (Vendola et al., 1999a,b). Likewise, pigs treated with the anti-androgen Flutamide reduced the mRNA and protein expression of IGF1R in the oocyte, and showed delayed primordial follicle activation (Knapczyk-Stwora et al., 2013). In preantral follicles isolated from women suffering from PCOS, an enhanced expression of IGF1R mRNA and protein was noted compared to controls (Stubbs et al., 2013). In the IGF-signaling system IGF binding proteins (IGFBPs) have in recent years received increased attention, because of their potential active modulating role of IGF-bioavailability. This is in contrast to the conventional idea about IGFBPs as simple carrier proteins. The IGFBPs bind IGF and sequester the binding of IGF to its receptors. This modulating role might be important in terms of shifting from the dormant to the activated follicular stage (Hu et al., 2017).

We hypothesize that primordial follicles may be androgen responsive based on the presents of components supporting androgen signaling, and that the action of androgens could be closely connected to the expression of key players in the IGF-signaling such as IGF1R, IGF2, and IGFBP3.

## Results

The global RNA transcriptomes representative for granulosa cells from primordial and primary follicles (http://users-birc.au.dk/biopv/published_data/ernst_et_al_GC_2017/) (Ernst et al., 2018) revealed 12.872 and 11.898 transcripts in granulosa cells from primordial and primary follicles, respectively (Ernst et al., 2018). The lists were further processed to exclude transcripts that were not consistently expressed in all patients and lists representative of stage-specific consistently expressed genes (SSCEGs) were generated. We applied this strict filter to only include analysis of genes that were consistent between patients included in this study, but certainly does not rule out that additional genes could be relevant. The SSCEGs analysis of the granulosa cell transcriptome revealed 1695 transcripts in primordial follicles and 815 transcripts in primary follicles. We further applied strict bioinformatic filters, and quality control to ensure specificity in output transcriptomes, and confirmed the presence of known granulosa cell-specific factors, as well as the absence of oocyte-specific factors. The SSCEGs lists in granulosa cells from primordial and primary follicles (Ernst et al., 2018) were used to extract genes differentially expressed genes (DEG) between the two cell populations.

### The “androgen signaling” pathway

We identified the most enriched and significant Canonical Pathways in granulosa cells from primordial and primary follicles (Ernst et al., 2018). To further analyze the ‘Androgen Signaling’ Pathways, we used the Ingenuity Pathway Analysis (IPA®) analysis software, which can be used to determine the most significant pathways and the genes allocated with each pathway. We found “Androgen Signaling” from Canonical Pathways significantly and differentially enriched in granulosa cells from both primordial and primary follicles (Table 1).

**Table 1:** “Androgen Signaling” pathway annotations—granulosa cells from primordial follicles.

**Gene name**	**Gene symbol**	**FPKM mean value**	***p*-value**
RNA Polymerase II Subunit D	*POLR2D*	2,508	0,186
G Protein Subunit Alpha 12	*GNA12*	1,573	0,195
G Protein Subunit Alpha Q	*GNAQ*	4,444	0,121
RNA Polymerase II Subunit J	*POLR2J*	2,812	0,066
General Transcription Factor IIH Subunit 2	*GTF2H2*	3,333	0,165
CDK Activating Kinase Assembly Factor	*MNAT1*	1,618	0,157
G Protein Subunit Alpha I2	*GNAI2*	1,574	0,184
G Protein Subunit Gamma 11	*GNG11*	1,521	0,038
Protein Kinase C Iota	*PRKCI*	4,140	0,027
Androgen Receptor	*AR*	3,134	0,012
Protein Kinase C Eta	*PRKCH*	3,709	0,172
G Protein Subunit Gamma 5	*GNG5*	3,200	0,003
Protein Kinase D3	*PRKD3*	1,538	0,174
Protein Kinase C Beta	*PRKCB*	2,157	0,112
Protein Kinase C Alpha	*PRKCA*	4,440	0,087

“*Androgens Signaling” pathway annotation of the 15 transcripts identified in granulosa cells from primordial follicles. FPKM mean values were calculated based on triplicate expression values of the same transcript using a one-sample t-test. The p-value is indicative of the consistency in expression pattern across triplicates*.

**Figure 1:**
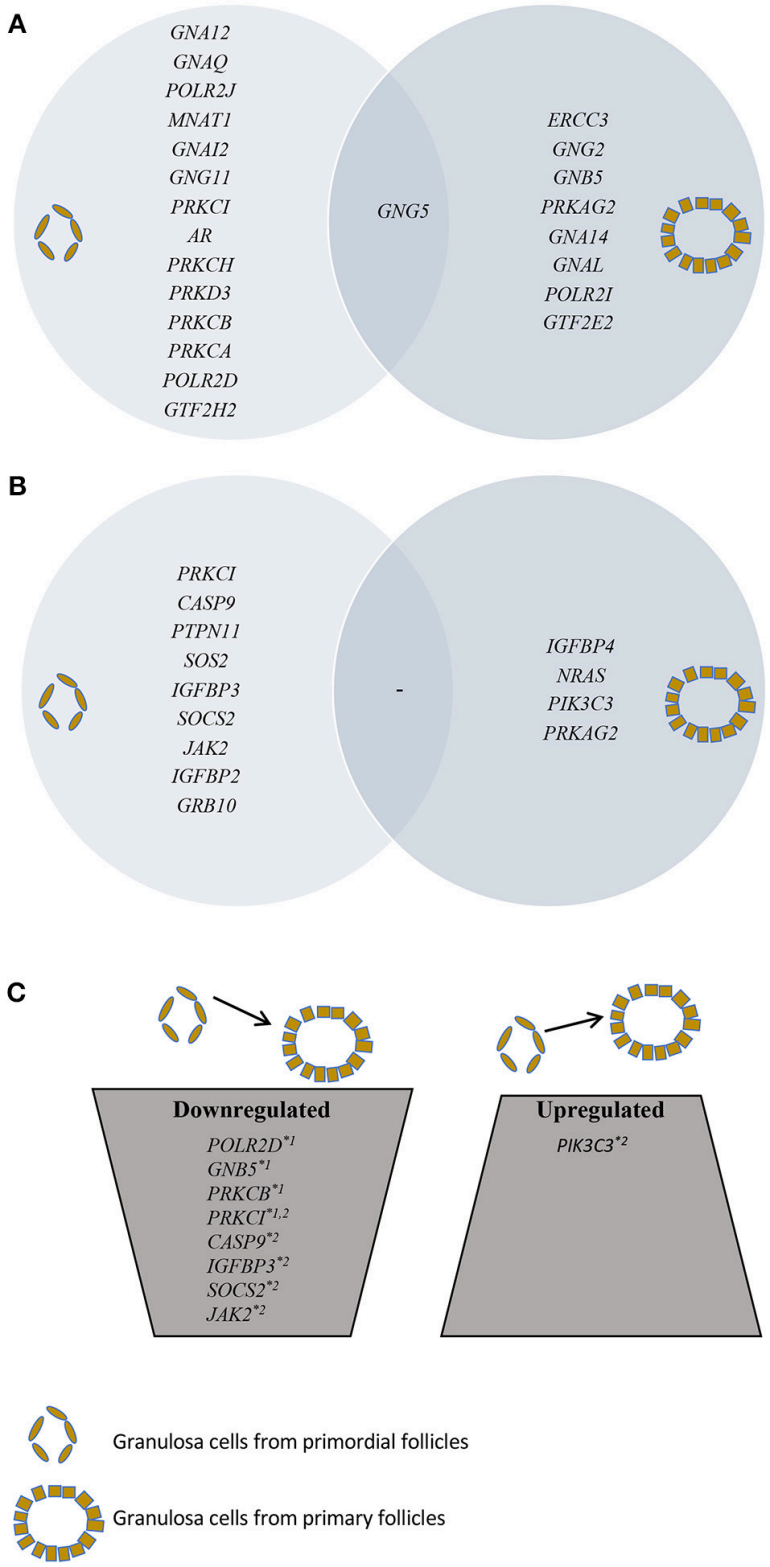
Presentation of SSCEGs and DEGs in granulosa cells from primordial and primary follicles. **(A)** Presentation of SSCEGs from the “AR Signaling” Pathway that are significantly expressed in granulosa cell from primordial and primordial follicles, as indicated, and the number in the middle representing SSCEG in both stages. **(B)** Presentation of SSCEGs from the “IGF1 Signaling” Pathway that are significantly expressed in granulosa cell from primordial and primary follicles, as indicated, and number in the middle representing SSCEG in both stages. **(C)** Presentation of DEGs from both “AR Signaling” (Noted ^*^1) and “IGF1 Signaling” (Noted ^*^2) pathways.

In granulosa cells from primordial follicles, the “Androgen Signaling” was highly enriched (*p* = 3,97E-02) with 15 genes assigned (*POLR2D, GNA12, GNAQ, POLR2J, GTF2H2, MNAT1, GNAI2, GNG11, PRKCI, AR, PRKCH, GNG5, PRKD3, PRKCB, PRKCA*), including the androgen receptor *(AR)* (Table 1; Figure 1A). The *AR* transcript is low-to-moderately expressed (mean FPKM value of 3.13) with a *p*-value of *p* = 0.012, indicating that the *AR* transcript is consistently expressed in the samples tested.

The “Androgen Signaling” was also enriched in granulosa cells from primary follicles (*p* = 3,42E-02) with nine genes (*POLR2I, ERCC3, GNG2, GNB5, PRKAG2, GTF2E2, GNA14, GNG5, GNAL*) assigned (Table 2; Figure 1A). In granulosa cells from primary follicles, the *AR* transcript levels is very low (mean FPKM value is 1.65) and was not consistently expressed in our samples (*p* = 0.39).

**Table 2:** “Androgen Signaling” pathway annotations—granulosa cells from primary follicles.

**Gene name**	**Gene symbol**	**FPKM mean value**	***p*-value**
RNA Polymerase II Subunit I	*POLR2I*	1,712	0,042
ERCC Excision Repair 3, TFIIH Core Complex Helicase Subunit	*ERCC3*	1,563	0,190
G Protein Subunit Gamma 2	*GNG2*	2,731	0,185
G Protein Subunit Beta 5	*GNB5*	0,912	0,193
Protein Kinase AMP-Activated Non-Catalytic Subunit Gamma 2	*PRKAG2*	3,106	0,012
General Transcription Factor IIE Subunit 2	*GTF2E2*	3,597	0,194
G Protein Subunit Alpha 14	*GNA14*	4,808	0,075
G Protein Subunit Gamma 5	*GNG5*	1,989	0,119
G Protein Subunit Alpha L	*GNAL*	4,123	0,187

*Androgens Signaling” pathway annotation of the nice transcripts identified in granulosa cells from primary follicles. FPKM mean values were calculated based on triplicate expression values of the same transcript using a one-sample t-test. The p-value is indicative of the consistency in expression pattern across triplicates*.

### Differentially expressed genes in the “androgen signaling” pathway

**Figure 2:**
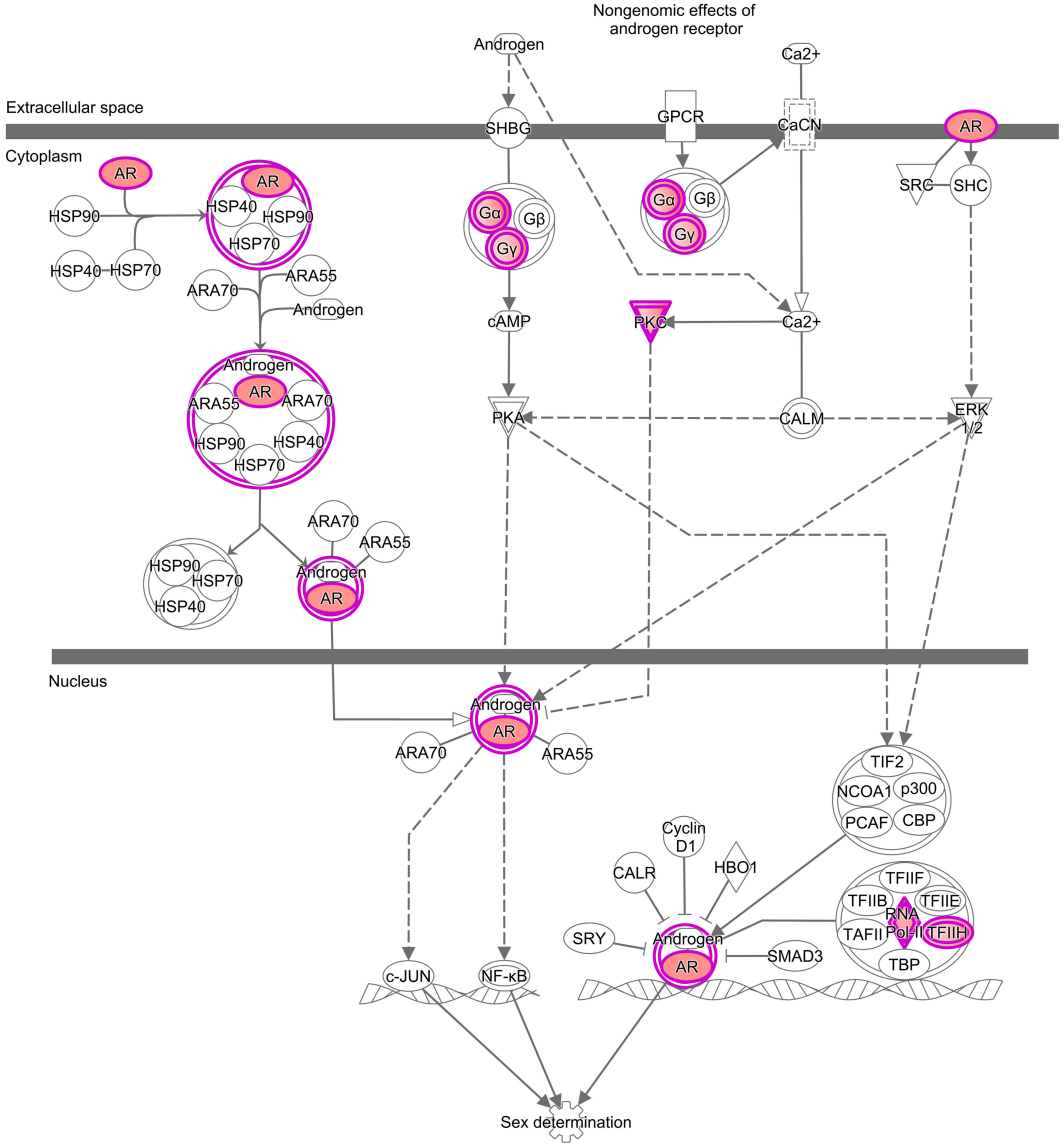
Enrichments analysis and IPA® pathways highlighting trends in granulosa cells from primordial and primary follicles, respectively. Pathway analysis (IPA^®^) of the “Androgen Signaling” pathway in granulosa cells of the primordial follicle. Color intensities are based on FPKM values where high significance is most intensive in color (red).

During the primordial to primary follicle transition, “Androgen Signaling” was non-significantly down-regulated (*p* = 5,65E-01). However, four genes (*PRKC1, POLR2D, GNB5*, and *PRKCB*) from the “Androgen Signaling” pathway were significantly down-regulated in the granulosa cells (Table 3; Figure 1C). As noted above, the *AR* transcript was down-regulated, however not significantly. The four genes were down-regulated by 2-fold change, indicating a rapid chance in expression during the primordial-to-primary transition. Interestingly, no genes from the androgen signaling pathway was significantly up-regulated during the primordial to primary follicle transition. The Androgen Signaling Pathway and the molecular network associated with this pathway is illustrated in Figure 2.

**Table 3:** Differently expressed genes annotated “Androgen Signaling” pathway.

**Gene Name**	**Gene Symbol**	**GC from PDF mean FPKM value**	***p*-value**	**GCs from PMF FPKM value**	***p*-value**	**Significance, paired *t*-test**	**Fold-change down**
Protein Kinase C Iota	*PRKCI*	4,140	0,027	1,999	0,410	0,226	2,071
RNA Polymerase II Subunit D	*POLR2D*	2,508	0,186	1,236	0,423	0,460	2,029
G Protein Subunit Beta 5	*GNB5*	2,043	0,211	0,912	0,193	0,426	2,242
Protein Kinase C Beta	*PRKCB*	2,157	0,112	1,239	0,236	0,010	1,741

*Differently expressed genes identified by comparing transcriptomes of the granulosa cells from primordial follicles vs. granulosa cells from primary follicles. Four genes were significantly downregulated during the primordial to primary follicle transition. Significance: fold-change >2 and/or paired t-test significance (p < 0.05) between the two FPKM mean values*.

### Differentially expressed genes in “IGF1 signaling” pathway

We interrogated the presence of *IGF1, IGF2, IGF1R*, and *IGFR2* as well as the *IGFBP1-6* transcripts in human oocytes and granulosa cells from primordial and primary follicles (Ernst et al., 2017, 2018) and found that some were significantly expressed, whilst close to be significant across the triplicates of samples (Table 4). It is noteworthy that the expression levels of *IGF2* and *IGF1* in granulosa cells from primordial cells to primary follicles decreased, while *IGF1R* and *IGF2R* expression levels remained. In oocytes from primordial and primary follicles, *IGF2*, and *IGF1R* transcripts increased, while *IGF1* and *IGF2R* transcript were not significantly altered. Interestingly, expression of *IGFBP1-6* varied significantly, with *IGFBP-5* being highly expressed in all cells from both primordial and primary follicles. The *IGFBP3* transcript appears to be upregulated in oocytes from primordial and primary follicles, but down regulated from granulosa cells from primordial cells to primary follicles.

**Table 4:** Expression of *IGF1, IGF1R, IGF2, IGF2R*, and *IGFBP1-6* transcripts.

	Oocytes^*^				Granulosa cells^*^			
	**Primordial follicles**		**Primary follicles**		**Primordial follicles**		**Primary follicles**	
**Gene names**	**FPKM means**	***p*-value**	**FPKM means**	***p*-value**	**FPKM means**	***p*-value**	**FPKM means**	***p*-value**
*IGF1*	2,696	0,25	1,928	0,404	2,826	0,222	1,767	0,018
*IGF1R*	4,610	0,091	7,582	0,017	7,758	0,016	7,149	0,018
*IGF2*	6,031	0,049	8,340	0,025	4,654	0,101	1,631	0,379
*IGF2R*	1,987	0,231	1,572	0,198	4,683	0,203	3,157	0,184
*IGFB1*	0,935	0,423	0,307	0,301	0,489	0,353	0,608	0,423
*IGFB2*	1,236	0,147	2,168	0,094	3,217	0,030	2,445	0,221
*IGFB3*	0,834	0,423	3,323	0,012	3,356	0,067	0,784	0,423
*IGFB4*	1,026	0,122	0,896	0,191	1,523	0,225	1,547	0,103
*IGFB5*	5,071	0,052	6,283	0,007	7,116	0,005	5,733	0,052
*IGFB6*	–	–	–	–	1,439	0,303	0,056	0,423

*Gene names are followed by the mean FPKM and p-values for human oocytes and granulosa cells from primordial and primary follicels, as indicated*.

* *Data extracted from Ernst et al. (2018) and Kuijjer et al. (2013)*.

Although the “IGF1 signaling” pathway was not significantly enriched in granulosa cells from primordial (*p* = 4,25E-01) or primary follicles (*p* = 5,56E-01), it was selected for further analysis, as IGFs are important for ovarian physiology (Adashi et al., 1985, 1991; Mondschein et al., 1989; Armstrong et al., 1996; Baumgarten et al., 2014). The IGF Signaling Pathway from the IPA® analysis contains factors directly associated with the IGF system (e.g., IGF1 and IGF2 and their respective receptors, IGF1 receptor and IGF2 receptor, as well as six binding proteins, IGFBP1-6) as well as the signal tranducting factors requires to conduct the IGF signaling (Laviola et al., 2007; Kuijjer et al., 2013; Lodhia et al., 2015), including PKC, Caspase9, JAK2, PIK3C3, and PRKCI. Consequently, it is noteworthy that many of the signal transducing components assigned to IGF Signaling Pathways are also found in other Signal transducing pathways, such as EGFR signaling. The “IGF-1 Signaling” pathway in the granulosa cells from primordial follicles contained nine genes (*PRKCI, CASP9, PTPN11, SOS2, IGFBP3, SOCS2, JAK2, IGFBP2, GRB10)* (Table 5; Figure 1B), and from primary follicles, we noted four genes (*IGFBP4, NRAS, PIK3C3, PRKAG2* (Table 6; Figure 1B), suggesting a dynamic change of “IGF1 signaling”-related genes during the primordial to primary transition. During the primordial to primary follicle transition, the ‘IGF1 Signaling’ pathway was non-significantly downregulated (*p* = 5,64E-01). Interestingly, during the primordial to primary follicle transition, several members of the IGF1 Signaling family were significantly down- or up-regulated. Five genes (*PRKC1, CASP9, IGFBP3, SOC2, JAK2*) (Figure 1C) were significantly downregulated during the transition, and one gene (*PIK3C3*) (Figure 1C) was significantly up-regulated (Table 7).

**Table 5:** “IGF1 Signaling” pathway annotations—GCs from primordial follicles.

**Gene name**	**Gene symbol**	**FPKM mean value**	***p*-value**
Protein Kinase C Iota	*PRKCI*	4,140	0,027
Caspase 9	*CASP9*	0,357	0,192
Protein Tyrosine Phosphatase, Non-Receptor Type 11	*PTPN11*	2,207	0,193
SOS Ras/Rho Guanine Nucleotide Exchange Factor 2	*SOS2*	2,635	0,006
Insulin Like Growth Factor Binding Protein 3	*IGFBP3*	3,356	0,067
Suppressor Of Cytokine Signaling 2	*SOCS2*	1,768	0,186
Janus Kinase 2	*JAK2*	5,792	0,022
Insulin Like Growth Factor Binding Protein 2	*IGFBP2*	3,217	0,030
Growth Factor Receptor Bound Protein 10	*GRB10*	2,420	0,191

“*IGF1 Signaling” pathway annotation of the nine transcripts identified in granulosa cells from primordial follicles. FPKM mean values were calculated based on triplicate expression values of the same transcript using a one-sample t-test. The p-value is indicative of the consistency in expression pattern across triplicates*.

**Table 6:** “IGF1 Signaling” pathway annotations—granulosa cells from primary follicles.

**Gene Name**	**Gene symbol**	**FPKM mean value**	***p*-value**
Insulin Like Growth Factor Binding Protein 4	*IGFBP4*	1,547	0,103
NRAS Proto-Oncogene, GTPase	*NRAS*	4,664	0,087
Phosphatidylinositol 3-Kinase Catalytic Subunit Type 3	*PIK3C3*	3,822	0,170
Protein Kinase AMP-Activated Non-Catalytic Subunit Gamma 2	*PRKAG2*	3,106	0,012

“*IGF1 Signaling” pathway annotation of the four transcripts identified in granulosa cells from primary follicles. FPKM mean values were calculated based on triplicate expression values of the same transcript using a one-sample t-test. The p-value is indicative of the consistency in expression pattern across triplicates*.

**Table 7:** Differently expressed genes annotated “IGF1 Signaling” pathway.

**Gene name**	**Gene Symbol**	GC^*from PDF mean FPKM value^	***p*-value**	GC^*^from PMF FPKM value	***p*-value**	**Significance, paired *t*-test**	**Fold-change down**
**SIGNIFICANTLY DOWN-REGULATED IN GRANULOSA CELLS DURING PRIMORDIAL TO PRIMARY TRANSITION**							
Protein Kinase C Iota	*PRKCI*	4,140	0,027	1,999	0,410	0,226	2,071
Caspase 9	*CASP9*	0,357	0,192	0	0	Significant	∞
Insulin Like Growth Factor Binding Protein 3	*IGFBP3*	3,356	0,067	0,784	0,423	0,003	4,280
Suppressor Of Cytokine Signaling 2	*SOCS2*	1,768	0,186	0,300	0,307	0,318	5,894
Janus Kinase 2	*JAK2*	5,792	0,022	1,436	0,423	0,123	4,034
**SIGNIFICANTLY UP-REGULATED IN GRANULOSA CELLS DURING PRIMORDIAL-PRIMARY TRANSITION**							
Phosphatidylinositol 3-Kinase Catalytic Subunit Type 3	*PIK3C3*	3,254	0,198	3,822	0,170	0,033	0,851

*Differently expressed genes identified by comparing transcriptomes of the granulosa cells from primordial follicles vs. granulosa cells from primary follicles. Five genes were significantly downregulated, and one gene was significantly upregulated during the primordial to primary follicle transition. Significance: fold-change >2 and/or paired t-test significance (p < 0.05) between the two FPKM mean values*.

* *Granulosa cells*.

### IGF2 protein localizes to human oocytes and granulosa cells in primordial and primary follicles

**Figure 3:**
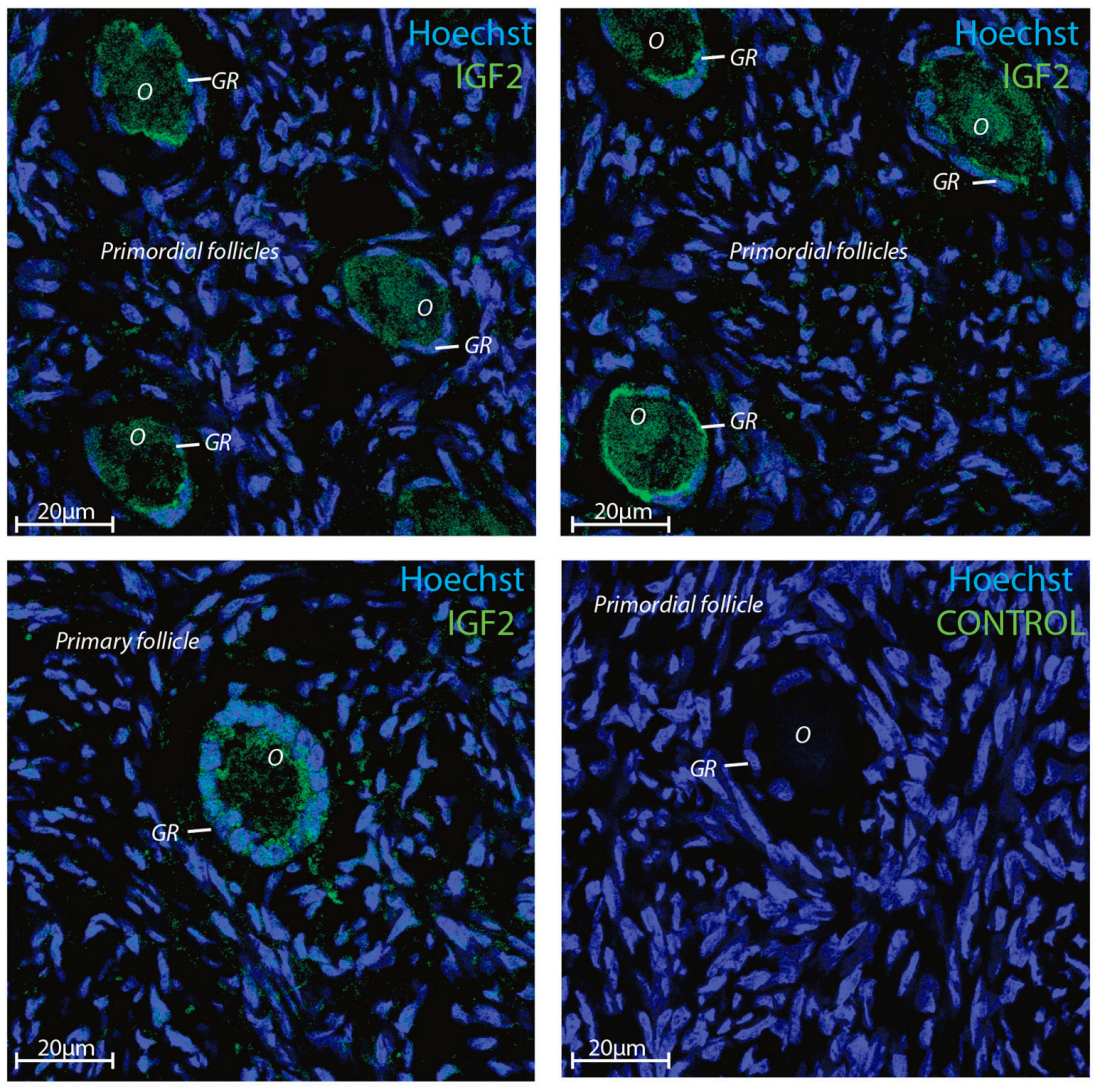
Intra-ovarian distribution of IGF2 in human granulosa cells from primordial and primary follicles. Images show that IGF2 localized to oocytes and granulosa cells in primordial and primary follicles. A control without primary IGF2 antibody was included and reveals no staining. Hoechst staining identifies the nucleus of cells. Scale bars; 20 μm.

Transcriptomic data represents RNA profiling and thus, does not necessarily represents protein expression profiles. Human oocytes are loaded with maternal mRNA, of which many are packed into a protein complex preventing translation at this stage. The IGF2 protein was selected for immunofluorescent staining since the *IGF2* gene was highly expressed in both oocytes and granulosa cells in primordial and primary follicles (Ernst et al., 2017, 2018) (Table 4). The immunofluorescence revealed a strong staining of the IGF2 protein in both oocytes and granulosa cells in primordial and primary follicles (Figure 3). The staining of IGF2 apeared as membranous and cytoplasmic staining in both oocytes and granulosa cells in primordial and primary follicles (Figure 3). The nuclear counter stain is Hoechst (blue) (Figure 3). Quantification of the IGF2 staining in primordial (pixel intensity = 16,1) and primary (pixel intensity = 20,7) follicles supports overall the RNA sequencing FPKM values noted for *IGF2* (Table 4), although distinction between cell compartments could not be precisely measured. As such, it is not possible to note of the upregulation of IGF2 is more strong in the oocytes compeared to the granulosa cells in the same follicels stages, although we observed that *IGF2* is uregulated on oocytes during the primordial to primary transition, in contrast to downregulation of *IGF2* in granulosa cells in the primordial to primary transition (Table 4).

### Several androgen-responsive genes appear to be expressed during the primordial to primary follicle transition

To further reveal a potential effect of androgen signaling in primordial and primary follicles, we interrogated the presence of known androgen response genes (Romanuik et al., 2009) in the global transcriptomes of oocytes (Ernst et al., 2017) and granulosa cells (Ernst et al., 2018) from primordial and primary follicles. Of the known androgen-responsive genes (87 genes), 62 genes were found present in the transcriptome data (Table 8). Several of the androgen-responsive genes were very highly expressed (*ABHD2, ATP1A1, B2M, FDFT1, GOLPH3, NDRG1, ODC1, PAK2, RPL15, SOD1, TCP1, TPD52*, and *TSC22D1*), and several moderately expressed (such as *ACSL3, ADAM28, CNBD1, DHCR24, MANEA, PIK3R3, TMEFF2*, and *USP33*).

**Table 8:** Expression of androgen-responsive genes.

Oocytes^*a^				Granulosa cells^*a^			
**Primordial follicles**		**Primary follicles**		**Primordial follicles**		**Primary follicles**	
**Gene names**	**FPKM means**	**Gene names**	**FPKM means**	**Gene names**	**FPKM means**	**Gene names**	**FPKM means**
***ABHD2***	**4,865**	***ABHD2***	**5,290**	***ABHD2***	**4,541**	***ABHD2***	**2,969**
***ACSL3***	**1,950**	***ACSL3***	**5,552**	***ACSL3***	**4,267**	*ACSL3*	2,909
***ADAM28***	**2,591**	*ADAM28*	3,043	***ADAM28***	**1,944**	*ADAM28*	2,500
*ADAMTS1*	0,057			*ADAMTS1*	1,10	*ADAMTS1*	0,967
*ARL6IP5*	0,196			*ARL6IP5*	2,712	*ARL6IP5*	0,332
***ATP1A1***	**4,888**	***ATP1A1***	**4,012**	***ATP1A1***	**4,514**	***ATP1A1***	**5,862**
***B2M***	**4,032**	***B2M***	**3,058**	***B2M***	**5,220**	***B2M***	**5,339**
				*BLVRB*	0,818	*BLVRB*	0,507
*C1orf21*	1,566	*C1orf21*	1,480	***C1orf21***	**4,202**	*C1orf21*	1,250
***CAMK2N1***	**0,323**	*CAMK2N1*	0,097	*CAMK2N1*	1,014		
*CAPNS1*	0,760	*CAPNS1*	0,118	*CAPNS1*	2,776	*CAPNS1*	0,406
						*C1orf216*	1,325
*CENPN*	0,123	*CENPN*	0,158	*CENPN*	0,060	*CENPN*	0,107
*CNBD1*	2,910	*CNBD1*	3,304	***CNBD1***	**3,958**	*CNBD1*	2,523
*DERA*	0,044	*DERA*	2,082	*DERA*	**1,649**	*DERA*	0,0564
***DHCR24***	**1,975**	*DHCR24*	0,968	***DHCR24***	**4,669**	*DHCR24*	2,454
*ENDOD1*	2,238	***ENDOD1***	**2,638**	***ENDOD1***	**2,235**	***ENDOD1***	**3,211**
***FDFT1***	**7,041**	***FDFT1***	**8,865**	***FDFT1***	**9,266**	***FDFT1***	**6,836**
*FKBP5*	0,897	*FKBP5*	1,859	*FKBP5*	0,681	*FKBP5*	1,671
***GOLPH3***	**2,791**	***GOLPH3***	**4,881**	***GOLPH3***	**4,029**	*GOLPH3*	2,701
***GOLPH3L***	**3,261**	*GOLPH3L*	1,225	***GOLPH3L***	**5,083**	***GOLPH3L***	**5,045**
		***HM13***	**3,727**	*HM13*	2,325	*KCNMA1*	1,842
		***HSP90B1***	**7,722**	***HSP90B1***	**7,403**		
*KCNMA1*	0,113	*KCNMA1*	0,061	*KCNMA1*	0,482		
				*KLK3*	1,962		
				*KRT8*	0,432	***KRT8***	**0,1900**
***LRIG1***	**1,775**	*LRIG1*	0,061	*LRIG1*	1,993	*LRIG1*	0,565
***MANEA***	**1,140**	*MANEA*	0,115	*MANEA*	1,158	*MANEA*	1,100
***NANS***	**0,152**			*NANS*	**3,206**	*NANS*	0,565
*NCAPD3*	1,729	*NCAPD3*	1,709	*NCAPD3*	**1,806**	*NCAPD3*	1,691
***NDRG1***	**3,044**	***NDRG1***	**4,234**	***NDRG1***	**5,523**	***NDRG1***	**3,950**
***NIPSNAP3A***	**2,785**	*NIPSNAP3A*	0,710	***NIPSNAP3A***	**2,661**	***NIPSNAP3A***	**1,870**
*NKX3-1*	0,057	*NKX3-1*	0,922	*NKX3-1*	0,2813	*NKX3-1*	0,866
*NTS*	0,098	*NTS*	0,097				
*NME7*	2,056	*NUCB2*	1,031	*NME7*	4,846	*NME7*	2,7182
*NUCB2*	0,7986			*NUCB2*	**3,498**	*NUCB2*	1,549
***ODC1***	**5,9116**	***ODC1***	**7,978**	***ODC1***	**6,689**	***ODC1***	**5,0963**
*OGDH*	1,692	*OGDH*	1,891	***OGDH***	**3,741**	***OGDH***	**2,950**
*PAK1IP1*	0,057	***PAK1IP1***	**4,685**	***PAK1IP1***	**2,872**	*PAK1IP1*	1,316
***PAK2***	**5,683**	***PAK2***	**5,029**	***PAK2***	**5,677**	***PAK2***	**5,630**
*PIK3R3*	1,872	*PIK3R3*	1,392	*PIK3R3*	2,114	*PIK3R3*	0,839
		*RHOU*	1,422	*RHOU*	0,927	*RHOU*	1,221
***PRKACB***	**0,152**			*PRKACB*	1,579	*PRKACB*	1,081
				*PPAP2A*	1,581		
				***RAB4A***	**2,826**		
***RPL15***	**5,512**	***RPL15***	**5,607**	***RPL15***	**6,169**	***RPL15***	**6,514**
***SEC61G***	**1,681**	***SEC61G***	**1,677**	***SEC61G***	**3,222**	***SEC61G***	**2,213**
***SF3B5***	**1,828**	***SF3B5***	**1,887**	***SF3B5***	**3,095**	***SF3B5***	**1,855**
*SLC41A1*	1,760	*SLC41A1*	0,993	*SLC41A1*	1,450	*SLC41A1*	1,496
*SLC45A3*	0,044	*SLC45A3*	0,097	*SLC45A3*	0,772		
***SOD1***	**6,402**	*SOD1*	4,241	***SOD1***	**7,034**	***SOD1***	**5,333**
***SORD***	**0,074**	*SORD*	1,069	***SORD***	**0,399**	*SORD*	1,301
						*STEAP4*	0,3615
***SVIP***	**3,027**	***SVIP***	**2,562**	***SVIP***	**2,701**	*SVIP*	3,008
***TAOK3***	**0,222**	*TAOK3*	0,506	***TAOK3***	**3,437**	*TAOK3*	1,203
***TCP1***	**5,834**	***TCP1***	**4,781**	***TCP1***	**4,7636**		
***TMEFF2***	**2,853**	***TMEFF2***	**1,049**	***TMEFF2***	**6,087**	*TMEFF2*	1,324
*TMPRSS2*	0,057			*TMPRSS2*	0,985		
***TPD52***	**3,712**	***TPD52***	**3,582**	***TPD52***	**5,543**	***TPD52***	**4,387**
*TPM1*	1,786	***TPM1***	**3,533**	*TPM1*	1,759	*TPM1*	3,031
***TSC22D1***	**4,368**	***TSC22D1***	**3,411**	***TSC22D1***	**4,891**	***TSC22D1***	**3,186**
***USP33***	**2,439**	***USP33***	**3,777**	***USP33***	**5,667**	*USP33*	2,556

*Gene names are followed by the mean FPKM values. Gene names and FPKM values in bold denotes SSCEGs*.

*a *Data extracted from Ernst et al. (2018) and (Kuijjer et al., 2013)*.

## Discussion

Ovarian follicles are subjected to to strict control of hormones and growth factors. In human granulosa cells, IGF1 is permissive for the positive feedback toward the FHS-induced expression of aromatase (CYP19A1) through AKT signaling (Baumgarten et al., 2014). This present study performed an *in silico* analysis of the transcriptomes representing granulosa cells from primordial and primary follicles, respectively. This provides a unique insight into the gene expression and perhaps actions of androgen-signaling and IGF-signaling in the two earliest stages of follicular development in the normal human ovary. Thus, we explored the potential of the earlist human follicles to be able to respond toward signals mediated by IGF- and androgen-signaling.

We applied strict filters in the bioinformatic management, and quality control to ensure the most precise outcome from the global transcriptome analysis. Therefore, the data presented must be evaluated with the fact that there is a fine balance between significant and non-significant outcomes. In some instances, variations between the data from the three patients is noted non-significant. Including more samples might even out this difference, and as many af of statistical analysis are close to a value for significance, it is most likely that most of the non-significant values indeed would be significant. The quantification of the IGF2 immunofluoresence on primordial and primary follicles aligned overall with the FPKM value, however, noteworthy, it is difficult to quantify immunofluorescent on slices performed on human follicles. Additionally, although maternally contributed mRNA are subjected to degradation, the turnover time for the corresponding protein may be differentially regulated and it is not known how much IGF2 protein that might be maternally supplied as well. Previous studies performed qPCR analysis to confirm the expression profiles of selected genes (Ernst et al., 2017, 2018) supporting that the FPKM values obtained reflects the intracellular levels. The analysis contains several DEG-lists based on both SSCEGs and non-SSCEGs. Therefore, caution in the analysis of fold of change for DEG transcripts is recommended. Importantly, this study interrogated the presence of transcripts, which do not necessarily reflect the corresponding protein product. Using single cell techniques, we are able to confirm the presence of proteins using immunohistochemistry. Interestingly, we found androgen signaling highly enriched in granulosa cells from primordial follicles and also enriched in granulosa cells from primary follicles, however less than in the granulosa cells from primordial follicles. Transcripts encoding for *AR* were significantly expressed in the granulosa cells from primordial follicles, and a non-significant downregulation of the *AR* gene expression in the granulosa cells during the primordial to primary follicle transition was detected, suggesting a dynamic expression of *AR* in the granulosa cells. This study is the first to show transcripts of *AR* expressed in the primordial follicle stage, which indicates an early responsiveness to androgens. Previously the *AR* transcript has been demonstrated in granulosa cells of rodent, primate and human from transitional follicles (oocyte, surrounded by one layer of mixed flattened and cuboidal granulosa cells) and onwards, but not in earlier follicular stages (Weil et al., 1999; Rice et al., 2007; Sen and Hammes, 2010). Interestingly, the androgen-responsive gene (*FDFT1*) encoding the Farnesyl diphosphate farnesyltransferase catalyzes the conversion of trans-farnesyl diphosphate to squalene, the first specific step in the cholesterol biosynthetic pathway, suggesting that already at the earliest stages of follicle development, the cells prepare to initiate steroidogenesis. The protein encoded by another androgen-responsive gene (*NDRG1*) appears to play a role in growth arrest and cell differentiation, possibly as a signaling protein shuttling between the cytoplasm and the nucleus. It is highly expressed during the primordial and primary transition, suggesting this candidate to be important for the activation of dormant oocytes. It is interesting that the androgen-responsive gene, the *SOD1* gene, encoding the superoxide dismutase-1 was highly expressed in both primordial and primary follicles. SOD1 is a major cytoplasmic antioxidant enzyme that metabolizes superoxide radicals to molecular oxygen and hydrogen peroxide, thus providing a defense against oxygen toxicity (Niwa et al., 2007). Intriguingly, the androgen-responsive gene, *PIK3R3*, encodes the phosphoinositide-3-Kinase Regulatory Subunit 3, a lipid kinases capable of phosphorylating the 3'OH of the inositol ring of phosphoinositide, and it has been demonstrated that IGF1R, INSR, and INSR substrate-1 (IRS1) bind to PIK3R3 *in vitro* (Dey et al., 1998). The study suggested that the interaction of PIK3R3 with IGFIR and INSR provides an alternative pathway for the activation of PI3-kinase.

This study interrogated the presence of the *AR* transcript through RNA sequencing during the human primordial to primary transition and suggests that at least parts of the AR responsive network, might be relevant during the first ovarian follicle activation step. Although a previous study did not detect the *AR* transcript in human primordial follicles (Suzuki et al., 1994; Rice et al., 2007), we believe this is attributable to technical limitations since that study used earlier version of mRNA preparations and RT-PCR analysis. The study further highlight the importance of interpretation of RT-PCR and point that its findings do not exclude the presence of a functionally active protein, and the possibility that androgens exert an effect from the earliest growing phase onwards (Rice et al., 2007). In human ovaries, AR was immunohistochemically localized to preantral, antral follicles, theca, and stroma (Chadha et al., 1994; Takayama et al., 1996; Saunders et al., 2000). However, the stage of preantral follicle development could not be specified due to the inherent insensitivity of these techniques. Interestingly in this regard, a study cultured porcine primordial follicles in the absence or presence of testosterone, and found that testosterone increased the activation of primordial follicles (Magamage et al., 2011). The study further utilized cyproterone acetate, an AR antagonist, which inhibited the stimulatory effect of testosterone on primordial follicle activation. In addition, the results from Western blot and immunohistochemistry also showed that the AR was present in porcine primordial follicles. The results from porcine primordial follicles suggest to the possibility that human early follicles may also contain AR protein, however, this remains to be established through immunohistochemistry and protein analysis. In oocytes from primordial follicles, our group has recently demonstrated low, inconsisten expression of *AR*, and no detectable expression in the oocyte of primary follicles (Ernst et al., 2017). The mechanism of action of androgens is primarily the direct activation of gene transcription, by binding of the ligand-receptor complex to androgen-response elements in the nucleus, but androgens are also known to induce more rapid non-genomic pathways via cytosolic AR and the MAPK/ERK pathway (Kousteni et al., 2001), and influence the IGF-signaling (Vendola et al., 1999a). In the transcriptomic data from granulosa cells from primordial follicles (Ernst et al., 2018), “IGF1 signaling” and “ERK signaling” were, however not significantly enriched, which suggests that the possible androgen signaling mechanisms in the granulosa cells from primordial follicles is based on binding of androgens to nuclear AR, and the direct genomic transcriptional induction. This is in contrast to the results from oocytes from primordial and primary follicles, where the “IGF1 Signaling” and “ERK signaling” pathways were both enriched (Ernst et al., 2017), demonstrating that the non-genomic cytosolic pathway might be the molecular mechanism of action of the androgens in the oocyte-compartment. Androgen signaling has besides the above-mentioned pathways also been linked to the canonical PI3K/PTEN/Akt pathway, which is known to regulate primordial follicle activation in human and rodent (Adhikari et al., 2012; Novella-Maestre et al., 2015). In neonatal mice, activation of the PI3K/PTEN/Akt pathway has been detected, with phosphorylation and translocation of FOXO3a, shortly after testosterone administration, as well as an increased percentage of growing follicles compared to controls (Yang et al., 2010). The link between androgen signaling and IGF1 signaling has been highly argued, as androgens were found to induce upregulation of IGF1 and IGF1R in oocytes of primordial follicles, which is positively correlated to follicular recruitment and activation (Vendola et al., 1999a). In the granulosa cells from primordial and primary follicles, we found that the “IGF1 Signaling” pathway as a group was not significantly enriched, but transcripts of several members of the IGF1 signaling family were detected including IGFBP3 and IGFBP2 in the primordial stage and IGFBP4 in the primary follicle stage. During the primordial to primary follicle transition IGFBP*3* was significantly down-regulated. We find this down-regulation of *IGFBP3* interesting, given IGFBP3's role as a modulator and antagonist of the IGF-IGFR interaction (Hu et al., 2017), which we speculate to be central in the fine-tuned regulation of IGF2-IGF1R interaction and thus downstream PI3K/PTEN/Akt activity in the activation of primordial follicles. Also, in the oocyte compartment, different interesting members of this pathway were detected including *IGF2* and *IGF1R*, both with a high expression (Ernst et al., 2017). Both the transcripts of *IGF1R* and *IGF2* were upregulated during the primordial to primary follicle transition in the oocyte, the latter of the two with a 2-fold increase. In contrast to the high expression of *IGF2* in oocytes from primordial and primary follicles, *IGF1* was only inconsistently expressed in a moderate level, which is consistent with previous studies on human ovarian tissue, showing that IGF2 seems more important than IGF1 in the normal ovarian physiology (Mazerbourg et al., 2003; Stubbs et al., 2013). However, our immunohistochemistry results clearly show that IGF2 protein is present in both oocytes and granulosa cell from primordial follicles, suggesting that it may also be maternally contributed as a protein. IGF1R is of particular interest, as it is a known upstream activator of the PI3K/PTEN/Akt pathway (Makker et al., 2014), which is known to be involved in the regulation of primordial follicles.

In the IGF signaling system, several other receptors besides IGF1R are also noteworthy; IGF2R and INSR. Transcripts of *IGF2R* and *INSR* were both detected in the oocytes, however the expression was low and inconsistent (Ernst et al., 2017). A higher expression of *IGF2R* was however detected in the whole follicle isolate compared to the oocyte only isolate, suggesting that *IGF2R* most likely is expressed in the granulosa cell compartment. Based on our collective results from the transcriptomic analysis of the primordial and primary granulosa cells and oocytes, and the existing literature, we pose the following hypothesis concerning the bidirectional communication in the primordial follicle activation: (1) Based on high *AR* expression, granulosa cells of primordial follicles may be androgen responsive through direct genomic action, (2) This responsiveness may induce transcription of paracrine factors, which in turn could stimulate the oocyte to express transcripts encoding IGF2 and IGF1R, (3) The regulation is the IGF signaling is tightly regulated, and the IGFBP1s are significant regulators of IGF signaling (Allard and Duan, 2018; Mazerbourg and Monget, 2018; Spitschak and Hoeflich, 2018). Therefore, the androgen responsiveness and its potential induction of *IGF2* and *IGF1R* transcription could be mediated through the activation of IGFBPs and IGF ligands in the grnaulosa cells during the primodial to primary transition, which through paracrine actions stimulate transcription of specific genes in the oocytes. In line with this, as mentioned above, androgen can mediate non-genomic signaling, which may also be relevant for IGF signaling. Activated AR in the cytoplasm can interact with several signaling molecules inclu ing the PI3K/Akt, Src, Ras-Raf-1, and PKC, which in turn converge on MAPK/ERK activation, leading to cell proliferation (Kamanga-Sollo et al., 2008). Cell signaling through androgen can also occur without ERK activation. Non-ERK pathways involve activation of mammalian target of rapamycin (mTOR) via the PI3K/Akt pathway or involvement of plasma membrane, G protein coupled receptors (GPCRs) and the sex hormone binding globulin receptor (SHBGR) that modulate intracellular Ca^2+^ concentration and cyclic adenosine monophosphate (cAMP) levels, respectively (Mellström and Naranjo, 2001; Heinlein and Chang, 2002). IGFBPs display higher binding affinities toward IGF than IGFR1/2, and IGFs are therefore regulated by IGFBPs (Firth and Baxter, 2002; Duan and Xu, 2005). During dormancy, granulosa cell-produced IGFBP3 could sequester IGF2 in the extracellular space, thus antagonizing ligand-receptor interaction. Upon IGFBP3-decrease in granulosa cells during the primordial to primary follicle transition, oocyte-derived IGF2 might be free to exert its local effect and to bind IGF1R on the oocyte thus stimulating growth in an autocrine manner, and at the same time bind to IGF2R on the granulosa cells to paracrinally stimulate cell growth, proliferation, differentiation, and/or survival. The scenario of IGF-mediated functions in ovarian physiology is intriguing and becomes very complex considering the pattern of *IGFBP* expression profiles (Mazerbourg and Monget, 2018). It has previously been reported that *IGFBP1* is expressed in granulosa cells of mature follicles (el-Roeiy et al., 1994; Kwon et al., 2010), and it is likely that the expression of *IGFBP1* increases during follicles development, as we note very low expression (if any) in both oocytes and granulosa cells from primordial and primary follicles. Previously, it was reported that *IGFBP2* decreases in the granulosa cells in an cAMP-dependent but FSH-independent manner, (Cataldo et al., 1993), suggesting perhaps an early role of this IGFBP in the early non-FSH responsive phase of follicle development. *IGFBP4* mRNA is expressed at low levels in both oocytes and granulosa cells from primordial and primary follicles, and is in line with a previous study that noted *IGFBP4* mRNA expression as decreasing during human follicle development (Kwon et al., 2010). While *IGFBP5* is higly expressed in all cells in both primordial and primary follicles, *IGPBP6* appears to be specific to granulosa cells in primordial follicles. Finally it is worthnoty that many IGF-independent functions have been reported for the IGFBPs (Allard and Duan, 2018), adding another layer of complexity to ovarian functions for this family, which hopefully will be addressed in future functional studies. Further studies are, however, needed to support this link between androgens and IGF-driven primordial follicle activation in the human ovary.

Androgens have received increased attention as a key-player in the early follicular development, as the hyperandrogenic microenvironment in the ovaries from women suffering from PCOS, is thought to be central in the anovulation phenotype (Franks and Hardy, 2010). Patients suffering from anovulatory PCOS is shown to have an increased percentage of growing follicles and stockpiling of the primary follicles compared to controls (Webber et al., 2003; Maciel et al., 2004). In a recent study, it was shown that oocytes from women suffering from hyperandrogenism have an increased expression of *IGF2* (Tian et al., 2017). In a future study comparing transcriptomic data from PCOS granulosa cells and oocytes, it would be interesting to investigate if also the *IGF1R* expression is increased in these patients. According to our hypothesis a potential pathogenic mechanism of PCOS could be that androgen-driven overexpression of *IGF1R*, would make the oocyte hypersensitive to growth factors such as IGF2, which is found in a high level, and thus trigger hyperactivity in the PI3K/PTEN/Akt pathway, resulting in a hyperactivation of primordial follicles.

## Materials and methods

### Tissue collection and follicle isolation

Normal ovarian cortex tissue was donated from three women undergoing oophorectomy followed by cryopreservation before gonadotoxic treatment of non-gynecological cancer. The patients were aged 26, 34, and 34 years old, respectively. Written informed consent was obtained from all patients. The study was approved by Danish Scientific Ethical Committee (Approval number: KF299017 and J7KF/01/170/99) and the Danish Data Protection Agency. From the donated random selected tissue pieces 539 primordial follicles and 261 primary follicles were collected using the Laser Capture Microdissection (LCM) technique using Veritas™ Microdissection Instrument Model 704 (Arcturus XT™, Molecular Devices, Applied Biosystems, Life Technologies, Foster City, CA, U.S.A). The follicles and oocytes were isolated based on their morphological appearance.

LCM, library preparation, sequencing, bioinformatics management, and enrichment analysis was performed essentially as described previously (Ernst et al., 2017, 2018). Briefly, thee human cortical fragments (2 × 2 × 1 mm) were thawed and fixed by immersion into 4% paraformaldehyde (PFA) at 4°C for 4 h followed by dehydration and embedment in paraffin, and the embedding and sectioning was performed as previously described (Markholt et al., 2012). For the LCM isolation, whole follicles and oocytes were captured based on morphological appearance. Oocytes surrounded by 3–5 flattened pre-granulosa cells were defined as primordial follicles, whereas primary follicles were identified as an oocyte surrounded by one layer of cuboidal granulosa cells. During the laser capture, an outline surrounding the cells of interest (oocyte only or whole follicles isolates) was marked microscopically and subsequently cut using the ultraviolet laser. Membrane glass slides (Arcturus® PEN Membrane Glass Slides, Applied Biosystems, Life Technologies, Foster City, CA, U.S.A.), enabled to lift the cells onto a sterile cap (Arcturus® CapSure® HS LCM Caps, Applied Biosystems, Life Technologies, Foster City, CA, U.S.A.) using infrared pulses.

RNA extraction, Library preparation and sequencing, mapping, and statistical analysis as previously described (Ernst et al., 2017, 2018). Briefly, Total RNA was extracted from LCM-isolated cells using Arcturus® Paradise® Plus RNA Extraction and Isolation Kit (#KIT0312I Arcturus Bioscience Inc., Mountain View, CA, U.S.A.), and subjected to linear amplification using the Ovation® RNA-Seq System V2 kit (NuGen Inc., San Carlos, CA, U.S.A.), and RNA-seq libraries were constructed from the output cDNA using Illumina TruSeq DNA Sample and Preparation kit (Illumina, San Diego, CA, USA), performed at AROS Applied Biotechnology, according to the manufacturer's protocol. BAM files were generated using Tophat (2.0.4), and Cufflinks (2.0.2) created a list of expressed transcripts. BWA (0.6.2) mapped all readings to the human reference genome (hg19). Expression of each gene in a given sample was normalized and transformed to a measurement of log2 [counts per million (CPM)]. Afterwards, fragments per kilobase of exon per million fragments mapped (FPKM) values were calculated on the basis of log2 (CPM) (R Core Team, 2012).

### Output from statistical analysis for enrichment analysis

*In silico* extraction of granulosa cell transcriptomes was performed on global transcriptome data from patient triplicates of oocytes and oocytes with surrounding granulosa cells (follicle) for both the primordial and primary stage (Ernst et al., 2017, 2018) applying strict filters. The FPKM for all detected transcripts was quantified by performing a *t*-test on patient triplicate samples of same type. The level of consistency was based on *p*-values, with a low *p*-value noting a high degree of consistency in FPKM mean across patient triplicates. The cut-off in the level of consistency for all transcripts was set at *p* < 0.2 across triplicates for being included in all downstream analyses. Afterwards, we identified transcripts uniquely detected in the follicle isolates, and not in corresponding oocytes. All transcripts with a value >1.5 FPKM, was considered uniquely expressed in the follicle isolates and regarded as granulosa cell transcriptome contributions.

Extraction of transcripts encoding the androgen receptor and IGF-related molecules was performed from the lists generated (Ernst et al., 2018) and shows SSCEG and DEG in granulosa cells from primordial (Tables 2, 3) and primary follicles (Tables 2, 5), respectively.

The canonical AR and IGF1 Signaling Pathways were built using IPA^®^ software (http://www.ingenuity.com).

### Immunofluorescence microscopy

Ovarian cortical tissue was sectioned in 5 μm slides and mounted on glass slides. Dehydration and antigen retrieval was performed as described elsewhere (Stubbs et al., 2005) followed by serum block (30 min), then primary antibody; anti-IGF2 rabbit polyclonal antibody (ab9574, Abcam, Cambridge, U.K.), (5 μg/ml) overnight at 4°C. This antibody was previously used and validated (Huang et al., 2010) and several other applications (http://www.abcam.com/igf2-antibody-ab9574-references.html). The sections were subsequently incubated in a 1:250 dilution of appropriate secondary antibody (donkey-anti-rabbit for IGF2) conjugated with Alexa Fluor 488 Dye (Life Technologies, Carlsbad, CA, U.S.A.). Sections were incubated in 1/3,500 Hoechst (Life Technologies, Carlsbad, CA, U.S.A.) followed by mounting with Dako Fluorescent Mounting Medium (Agilent Technologies, Santa Clara, CA, U.S.A) and analyzed using a LSM510 laser-scanning confocal microscope using a 63x C-Apochromat water immersion objective NA 1.2 (Carl Zeiss, Göttingen, Germany). Zen 2011 software (Carl Zeiss, Göttingen, Germany) was used for analysis and image capturing. The quantification of IGF2 immunofluorescence was done by as ImageJ (Jensen, 2013).

## Author contributions

LS, EHE, and KL-H conceived the study. LS, EHE, and KL-H analyzed NGS and IPA® data. MA performed ICH and analyzed data. EE provided ovarian tissue from patients. LS, EHE, and KL-H wrote the manuscript. All authors approved the final manuscript.

### Conflict of interest statement

The authors declare that the research was conducted in the absence of any commercial or financial relationships that could be construed as a potential conflict of interest.
